# Role of Inflammatory Biomarkers in Peripheral Arterial Disease: A Comprehensive Review of Prognostic and Therapeutic Implications

**DOI:** 10.3390/biom16060789

**Published:** 2026-05-27

**Authors:** Andreea Tudurachi, Bogdan-Sorin Tudurachi, Larisa Anghel, Radu Andy Sascău, Mircea Ovanez Balasanian, Cristina Prisacariu, Amin Bazyani, Cristian Stătescu

**Affiliations:** 1 “Grigore T. Popa” University of Medicine and Pharmacy, 700503 Iași, Romania; leonte.andreea@d.umfiasi.ro (A.T.); bogdan-sorin.tudurachi@d.umfiasi.ro (B.-S.T.); radu.sascau@umfiasi.ro (R.A.S.); ovanes718@yahoo.com (M.O.B.); cprisacariu88@gmail.com (C.P.); cristian.statescu@umfiasi.ro (C.S.); 2 Cardiology Department, Cardiovascular Diseases Institute “Prof. Dr. George I. M. Georgescu”, 700503 Iași, Romania; aminbazyani@gmail.com

**Keywords:** peripheral artery disease, chronic limb-threatening ischemia, inflammation, atherosclerosis, biomarkers, prognostic, anti-inflammatory therapy

## Abstract

**Background:** Peripheral artery disease (PAD) is a manifestation of systemic atherosclerosis characterized by chronic inflammation, endothelial dysfunction, and high residual risk of major adverse cardiovascular events (MACEs) and major adverse limb events (MALEs). This review aimed to summarize the prognostic role of inflammatory biomarkers in PAD and to discuss their therapeutic implications. **Methods:** A comprehensive narrative review was performed using PubMed/MEDLINE, Scopus, Web of Science, and Cochrane Library, focusing mainly on English-language studies published in recent years. Randomized trials, observational studies, systematic reviews, and meta-analyses evaluating inflammatory biomarkers and anti-inflammatory or vasculoprotective therapies in PAD were included. **Results:** Both classical and emerging inflammatory biomarkers were associated with PAD severity and adverse outcomes. C-reactive protein, fibrinogen, interleukins, tumor necrosis factor-α, myeloperoxidase, galectin-3, and growth differentiation factor-15 showed prognostic value for MACEs, MALEs, restenosis, amputation, and mortality. Among newer indices, the neutrophil-to-lymphocyte ratio, platelet-to-lymphocyte ratio, systemic immune-inflammation index, C-reactive protein-to-albumin ratio, and HALP (Hemoglobin, Albumin, Lymphocyte, and Platelet) score appear especially promising for risk stratification. Anti-inflammatory and pleiotropic therapies, including canakinumab, colchicine, statins, and PCSK9 (proprotein convertase subtilisin/kexin type 9) inhibitors, may help reduce residual inflammatory risk. **Conclusions:** Inflammatory biomarkers may improve prognostic stratification and support more personalized management in PAD. Their integration into clinical practice could enhance limb preservation and long-term cardiovascular outcomes.

## 1. Introduction

Cardiovascular diseases (CVD) are a major cause of premature death and place a significant burden on public health systems, representing a third of all global fatalities. PAD, stemming from atherosclerotic obstructions in the lower extremities, impacts more than 200 million individuals and is particularly common among the elderly; approximately 10% of those under 70 and 20% of those over 70 are affected. PAD serves as an indicator of systemic atherosclerosis, resulting in diminished blood flow, intermittent claudication (IC), and potentially chronic limb-threatening ischemia (CLTI), thereby elevating the likelihood of amputation [1,2,3,4,5].

**Figure 1:**
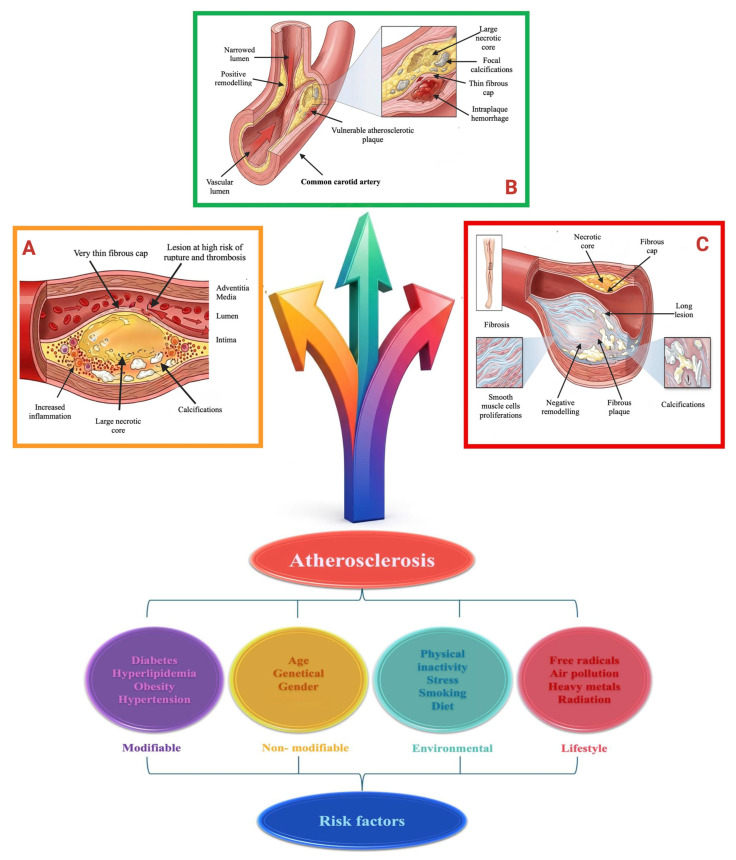
Atherosclerotic Plaque Morphology: A Comparative Anatomical Overview. (**A**). Coronary plaque morphology; (**B**). Carotid plaque morphology; (**C**). Peripheral artery plaque morphology.

PAD is primarily caused by factors such as high blood pressure, diabetes, high cholesterol, obesity, inflammation, and lifestyle choices. Over 60% of PAD patients present with atherosclerotic changes in other vascular regions, indicating that PAD is fundamentally a systemic atherosclerotic condition. Despite managing risk factors, residual cardiovascular issues and mortality remain high among PAD patients. Atherosclerosis is the main pathological process affecting the aorta and its branches, often leading to stenosis or aneurysms, with inflammation playing a significant role in the progression of both atherosclerosis and PAD [6,7]. Therefore, atherosclerosis is more than just a simple lipid-storage condition; it is a systemic, chronic inflammatory disease. Although atherosclerosis is shaped by common risk factors, the physical properties of plaques and the remodeling of arteries differ markedly among various arterial beds (Figure 1). Thin-Cap Fibroatheromas (TCFAs) are plaques that are more likely to form in coronary arteries. These plaques have a large necrotic core full of lipids, a lot of macrophages (foam cells) that have migrated into the plaque, and a very thin fibrous crown (defined as <65 µm). The main cause of acute coronary events is the rupture of the thin cap, which leads to acute local thrombotic occlusion. There is a strong link between fibroatheromatous plaques in the coronary system and positive remodeling. The vessel wall stretches outward to make room for plaque accumulation. This lets the vessel expand without affecting the luminal area until the disease reaches an advanced stage [8,9,10,11].

Carotid plaques are frequently more lipid-rich than coronary plaques; however, they generally possess a thicker fibrous cap (vulnerability is defined as <165 µm) and exhibit a diminished incidence of plaque erosion. Intraplaque hemorrhage (IPH) and plaque ulceration are major signs of vulnerability in the carotid bed. These happen more often here than in coronary arteries due to localized flow dynamics, which can lead to increased turbulence and shear stress that contribute to the formation of these vulnerable plaques. Carotid TCFAs and burst plaques, on the other hand, usually cause downstream embolic events like ischemic strokes. This phenomenon is different from coronary TCFAs, which cause local blockage. Carotid arteries, like coronary arteries, have outward positive remodeling to keep the artery lumen open. Therefore, wall thickness is often a better sign of high-risk plaque than the amount of stenosis itself [8,12,13,14].

In the lower limbs, especially in the femoropopliteal area, lesions are mostly fibrotic and heavily calcified. They have lower levels of lipids, fewer inflammatory foam cells, and more vascular smooth muscle cells. Because they are fibrotic, peripheral plaques are usually stronger and less likely to rupture than coronary plaques. Acute limb ischemia (ALI) is often ascribed to luminal thrombi in regions devoid of significant atherosclerosis, suggesting embolic origins or in situ thrombosis. Additionally, widespread medial arterial calcification (MAC) is a hallmark of peripheral disease, leading to inflexible, non-compliant arteries. The fibrotic properties of femoral plaques, in contrast to coronary arteries, are linked to constrictive (negative) remodeling, which directly diminishes the arterial lumen and results in increased restenosis rates post-treatment [8,10,12,15].

Plaques in the abdominal aorta are typically fibrotic and very calcified. They are mostly stable and are more likely to be involved in embolic events than thrombotic occlusions [16]. Renal artery stenosis exhibits a significant prevalence of pathological intimal thickening, reduced vasa vasorum, and a diminished area of dense calcium compared with coronary arteries. Lesions in the mesenteric arteries are mainly fibrotic [8,12].

Atherosclerosis is linked to endothelial dysfunction, oxidative stress, and metabolic changes. Oxidized low-density lipoproteins (LDL) induce inflammation and oxidative stress in blood vessel walls, prompting immune cells like monocytes to convert to macrophages and foam cells. This ongoing accumulation fosters chronic inflammation and the formation of atherosclerotic plaques, which can damage endothelial cells and create unstable plaques, leading to narrowed arteries and adverse outcomes such as chronic limb-threatening ischemia. Consequently, the development of predictive inflammatory biomarkers is essential in modern medical practice [1,6,7,17,18].

Recent advancements in PAD highlight the need for specific biomarkers to improve clinical assessment and prognosis, contrasting with coronary artery disease, where troponin is a well-established biomarker. Multiple classical biomarkers have been evaluated in clinical trials, providing vital diagnostic and prognostic information for PAD, although they are not routinely used in clinical practice. Contemporary investigations have also identified various non-conventional biomarkers that possess prognostic value, such as Soluble Suppression of Tumorigenicity 2 (sST2), Galectin-3, interleukins, matrix metalloproteinases, tumor necrosis factor-α (TNF-α), Lipocalin-2 (LCN-2), calprotectin, plasma pentraxin-3, and microRNAs. Despite this, their utilization within clinical practice is limited by high costs and the necessity for specialized data analysis [17,18].

**Figure 2:**
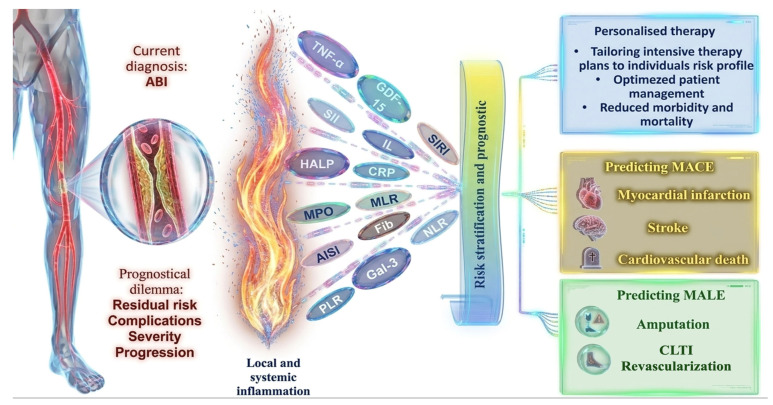
From Prognostic Dilemma to Tailored Management: The Clinical Utility of Inflammatory Biomarkers. The prognosis for PAD is complicated by several factors, including a significant ongoing cardiovascular risk and the presence of systemic atherosclerosis. Unlike coronary artery disease, PAD lacks specific biomarkers that would allow for effective screening. Moreover, the results of revascularization procedures can vary, especially for people with diabetes or chronic limb-threatening ischemia. The unique characteristics of lower limb plaque contribute to higher rates of restenosis after treatment. Current research concentrates on classical and modern inflammatory biomarkers to evaluate systemic inflammation, endothelial dysfunction, and thrombotic activity. Combining these biomarkers with traditional clinical risk factors could improve early risk assessment. This, in turn, would help create personalized treatment plans aimed at reducing death and illness. ABI—Ankle-Brachial Index; AISI—The Aggregate Index of Systemic Inflammation; CRP—C-Reactive Protein; Fib—fibrinogen; Gal-3—Galectine-3; GDF-15—Growth Differentiation Factor 15; HALP score—Hemoglobin, Albumin, Lymphocyte, and Platelet score; IL—interleukins; MPO—myeloperoxidase; MLR—monocyte/lymphocyte ratio; NLR—Neutrophil-to-Lymphocyte Ratio; PLR—Platelet-to-Lymphocyte Ratio; SII—Systemic Immune-Inflammation Index; SIRI—Systemic Inflammatory Response Index; TNF-α—Tumor Necrosis Factor-α.

Biomarkers are essential in modern medicine, significantly improving how we assess a patient’s risk of serious complications. This is especially important in diseases like PAD, which often do not show symptoms early on. They address the limitations of traditional diagnostic methods by revealing hidden biological factors, predicting negative outcomes, and supporting personalized treatment through precise risk assessment. To be effective, biomarkers must exhibit reliability, reproducibility, independence, accuracy, specificity, accessibility, cost-effectiveness, and the capacity for dynamic profiling. Conventional PAD evaluations, such as the ankle-brachial index and angiography, primarily focus on anatomical and hemodynamic factors, overlooking biological influences, which can lead to variable patient outcomes. As a result, biomarkers are crucial for assessing inflammation and identifying patients at heightened risk for cardiovascular and limb-related complications, thus facilitating a shift from reactive to proactive management approaches (Figure 2) [18,19,20].

The present manuscript provides a timely and comprehensive response to a critical bottleneck in vascular disease management, distinguishing itself from recent descriptive literature through its highly integrative structure. Existing publications often fragment the problem of vascular inflammation, addressing either basic plaque biology, isolated blood parameters, or emerging drug trials in an uncoordinated manner. Our manuscript explicitly rejects this siloed approach by constructing a cohesive, single-source narrative that systematically links the microscopic landscape of PAD, including anatomical disparities in lower-limb plaque stability, to macroscopic outcomes such as MACEs, MALEs, restenosis, amputation, and long-term mortality. By contextualizing both classical and modern hematological markers not merely as static diagnostic metrics but as dynamic indicators of an actively modifiable “residual inflammatory risk,” this review serves as a unified translational tool. Therefore, this review aims to move beyond a descriptive biomarker catalog and to provide an updated synthesis relevant to precision risk stratification and individualized PAD management. In doing so, it updates the state of vascular science up to 2026 and shifts the clinical conversation from reactive management toward a proactive, precision-driven therapeutic strategy.

Thus, the aim of this review is to provide a comprehensive and clinically relevant overview of the role of inflammation in peripheral artery disease, with particular emphasis on inflammatory biomarkers for risk stratification and on medical therapies targeting vascular inflammation. By clarifying the prognostic significance of inflammatory activity in PAD, this review highlights the potential of biomarker-guided assessment to identify high-risk patients earlier, refine individualized therapeutic strategies, and improve clinical decision-making. A more precise characterization of inflammatory burden may ultimately contribute to better cardiovascular and limb-related outcomes, including reduction in MACEs, MALEs, restenosis, and amputation risk.

## 2. Materials and Methods

**Figure 3:**
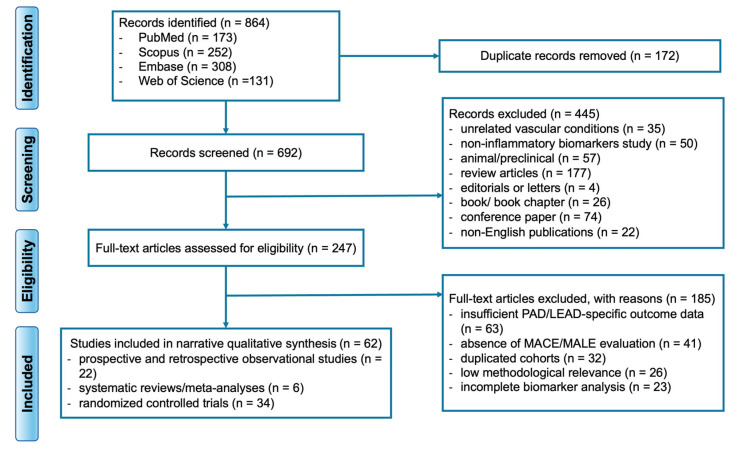
Preferred Reporting Items for Systematic reviews and Meta-Analysis (PRISMA) diagram.

Although the present manuscript was designed as a comprehensive narrative review rather than a formal systematic review or meta-analysis, a PRISMA 2020-style approach was applied to enhance transparency in the literature selection process. The identification, screening, eligibility assessment, and inclusion of studies were summarized using a structured flow diagram to improve methodological clarity and reproducibility (Figure 3). A thorough examination of existing scholarship was undertaken, drawing upon primary electronic medical databases including PubMed/MEDLINE, Scopus, Web of Science, and the Cochrane Library, to pinpoint pertinent studies published mainly within the preceding five years. The search methodology incorporated a blend of Medical Subject Headings (MeSH) and free-text keywords, such as “Peripheral Artery Disease”, ”Chronic Limb-Threatening Ischemia”, “inflammation,” “atherosclerosis,” “biomarkers,” “Neutrophil-to-Lymphocyte Ratio”, “C-reactive protein”, “lower-extremity atherosclerotic arterial disease” and pharmacological interventions like “colchicine,” “statins,” “PCSK9 inhibitors,” and “SGLT2 inhibitors.” This review focused on English-language, peer-reviewed publications. These included randomized controlled trials (RCTs), observational studies (both prospective and retrospective), systematic reviews, and meta-analyses. Studies were included if they assessed the prognostic significance of systemic inflammatory biomarkers or investigated the impact of medical treatments on MACEs and MALEs within populations diagnosed with PAD. For consistency with the established literature, PAD is defined herein specifically as lower-extremity atherosclerotic arterial disease. Titles and abstracts underwent independent screening for pertinence, followed by thorough full-text evaluations of studies that fulfilled the established criteria. Exclusions encompassed case reports, editorials, publications not in English, and studies presenting insufficient or duplicated data. Data synthesis was achieved through a narrative methodology, thereby enabling an evaluation of the fundamental inflammatory mechanisms implicated in PAD, the clinical value of particular biomarkers for risk assessment, and the efficacy of both established and novel atheroprotective approaches.

## 3. Classical Inflammatory Biomarkers

### 3.1. C-Reactive Protein (CRP)

CRP is a well-established biomarker commonly utilized to evaluate the systemic inflammatory burden. In patients with PAD, elevated baseline CRP levels serve as independent predictors of MACEs—comprising myocardial infarction (MI), stroke, and cardiovascular death), MALEs (defined as the need for revascularization or amputation), and short-term mortality. Furthermore, increased CRP concentrations correlate with advancing disease severity, as evidenced by a declining ABI and impaired walking capacity. In elderly individuals with PAD and type 2 diabetes mellitus, CRP (hazard ratio (HR): 1.5, 1.2–2.0) remained an independent predictor of mortality after adjusting for cardiovascular risk factors and duration of diabetes. A recent meta-analysis confirmed that higher initial CRP levels are significantly associated with poorer clinical outcomes following lower limb revascularization procedures [21,22,23]. Similarly, a prospective study by Nardella et al. demonstrated, in a multivariate logistic regression analysis, a direct correlation between elevated baseline CRP levels and adverse macrovascular complications, including both MACEs and MALEs (*p* < 0.01), in diabetic patients presenting with chronic limb-threatening ischemia (CLTI) (Table 1) [24]. Other studies examined risk factors for major amputations in patients with diabetic ulcers and CLTI receiving autologous cell therapy. Patients with major amputations showed significantly higher CRP levels (22.7 mg/L) compared to the group without major amputation (10.7 mg/L). Beyond standard assays, high-sensitivity CRP (hs-CRP) can detect significantly lower inflammatory thresholds, making it highly useful for identifying subclinical systemic vascular inflammation and refining cardiovascular risk stratification. While elevated pre-procedural hs-CRP levels are associated with a nearly 3-fold increase in all-cause mortality and a 4-fold higher risk of adverse limb outcomes in patients with PAD, lower baseline concentrations (<6.0 mg/L) correlate with superior limb salvage rates, highlighting the nuanced prognostic role of active vascular inflammation in limb-related survival. According to a recent meta-analysis of 13 studies evaluating the association between hs-CRP levels and cardiovascular outcomes in patients with PAD, elevated hs-CRP was associated with a significantly higher risk of all-cause mortality, demonstrating a pooled risk ratio of 3.49 (2.35–5.19) [25,26,27,28,29,30].

### 3.2. Fibrinogen (Fib)

Elevated levels of fibrinogen, an essential acute-phase reactant, serve as strong predictors of ischemic stroke, major bleeding events, limb amputation, and all-cause mortality, as well as the loss of primary patency following peripheral revascularization procedures [3,25,26,31,32]. Recent prospective data suggest that CLTI is characterized by a systemic inflammatory milieu that significantly subsides following the successful resolution of ischemia, underscoring the critical clinical importance of prompt therapeutic intervention. Active CLTI patients demonstrate a profound systemic inflammatory response, marked by elevated serum levels of positive acute-phase proteins (such as CRP and fibrinogen) coupled with reduced concentrations of negative acute-phase reactants, including albumin, total cholesterol, and high-density lipoprotein. This distinct inflammatory phenotype may be partially alleviated post-revascularization, thereby potentially mitigating its adverse systemic effects. In a prospective observational study involving 116 patients, Ferreira et al. revealed that individuals with CLTI exhibited elevated blood fibrinogen levels compared to those without CLTI (466.18 ± 208.07 mg/dL vs. 317.37 ± 79.42 mg/dL, *p* = 0.000). Moreover, CLTI was closely correlated with a progressive decrease in skeletal muscle mass, a pathological feature that independently increases the probability of major adverse cardiovascular events and severely reduces survival rates in individuals diagnosed with PAD [33].

### 3.3. Interleukins

Interleukin-1β (IL-1β), a potent pro-inflammatory cytokine, is strongly associated with the progression and instability of atherosclerotic plaques. Elevated baseline levels are predictive of poorer long-term cardiovascular outcomes. Specifically, in patients with diabetes and concurrent CLTI undergoing lower limb angioplasty, a multivariate logistic regression analysis demonstrated that higher preoperative concentrations independently predict an increased risk of major adverse MACEs and major amputations within one-year post-intervention. Moreover, elevated levels of the receptor antagonist may serve as a marker for a higher probability of disease recurrence or the development of de novo lesions within 12 months following surgical intervention [24,34].

Interleukin-6 (IL-6), another pivotal pro-inflammatory cytokine, plays a crucial role in orchestrating both the innate immune response and the chronic systemic inflammatory cascade. Elevated IL-6 levels are robustly linked to a higher incidence of MALEs and MACEs in patients with PAD. Specifically, Nardella et al. conducted a study with 264 patients, showing that higher IL-6 levels are linked to worse outcomes in diabetic patients with critical ischemia after revascularization. Furthermore, in diabetic cohorts, this cytokine serves as a reliable predictor of adverse postoperative outcomes following advanced revascularization procedures [24,25,26].

Interleukin-8 (IL-8), a pro-inflammatory cytokine, is significantly linked to advanced PAD and correlates with markedly elevated serum concentrations in patients presenting with CLTI, as demonstrated in a prospective, observational study of 119 patients, which included a cohort of 45 individuals with CLTI. High baseline and postoperative IL-8 levels are independently associated with diminished amputation-free survival (AFS). However, these elevated concentrations do not demonstrate a strong correlation with restenosis rates at six months following lower limb angioplasty [26,33].

Interleukin-10 (IL-10) is a pivotal anti-inflammatory cytokine that counter-regulates immune responses by inhibiting the production of pro-inflammatory cytokines. Elevated baseline IL-10 levels in diabetic patients with CLTI are paradoxically linked to worse clinical outcomes, including a higher risk of new lesion development or disease recurrence within 12 months post lower limb angioplasty or surgical interventions. Statistical analyses indicate that increased IL-10 levels significantly heighten risk, demonstrating a Hazard Ratio of 42.136 (*p* = 0.002), marking it as a critical predictive factor. Conversely, a dedicated study in patients with PAD demonstrated no significant correlation between mid-term IL-10 concentrations (at six months) and the incidence of in-stent restenosis [26,34].

Interleukin-27 (IL-27) is a dual-function cytokine capable of exerting both pro- and anti-inflammatory activities. It orchestrates this immunomodulatory balance by promoting the production of pro-inflammatory cytokines, such as IL-1 and tumor necrosis factor-alpha (TNF-α), while concurrently downregulating Th2 cytokines and upregulating interleukin-10. Recent evidence indicates that it is actively implicated in the pathophysiology of atherosclerosis, demonstrating a significant association with an elevated risk of MACEs and MALEs in patients with PAD undergoing revascularization. Notably, after multivariable adjustment for established cardiovascular and PAD risk factors, elevated values remained an independent predictor of MACEs (HR: 2.95; *p* = 0.039), although it did not maintain statistical significance for MALEs. Taken together, these findings indicate that increased baseline levels are associated with an unfavorable long-term cardiovascular prognosis [35].

### 3.4. Tumor Necrosis Factor-α

TNF-α is a major pro-inflammatory cytokine that exerts a profound impact on the vascular and skeletal muscle complications observed in patients with PAD. High circulating levels of TNF-α are robustly linked to adverse cardiovascular outcomes and an elevated risk of 12-month all-cause mortality; specifically, cumulative mortality at one year has been significantly associated with a TNF-α threshold of ≥8.1 (*p* = 0.048). Certain studies suggest that elevated perioperative TNF-α levels—both prior to and following intervention—may serve as reliable predictors of vein graft occlusion and revascularization failure, particularly in patients with coexisting diabetes, PAD, and CLTI undergoing endovascular procedures. Conversely, other investigations indicate no significant correlation between TNF-α concentrations and the incidence of in-stent restenosis following lower limb angioplasty [26,36,37].

### 3.5. Myeloperoxidase (MPO)

MPO, an enzyme primarily secreted upon neutrophil activation, exacerbates endothelial dysfunction, plaque instability, and atherosclerosis by generating reactive oxidants and diminishing nitric oxide bioavailability. Patients with chronic PAD exhibit significantly elevated circulating levels of MPO, which correlate with adverse cardiovascular and limb-related outcomes. Specifically, high baseline MPO levels are predictive of acute cardiovascular events and MALEs at six months follow-up. Receiver operating characteristic (ROC) curve analysis demonstrates that MPO possesses a fair prognostic performance for these outcomes, yielding an area under the curve (AUC) of 0.74 (CI: 0.56–0.91), with a sensitivity of 0.80 and a specificity of 0.65 at a validated cutoff value of 108.37 ng/mL. Furthermore, MPO serves as an independent predictor of MACEs, although it does not demonstrate a strong correlation with the baseline clinical severity of chronic PAD [38,39].

### 3.6. Galectin-3 (Gal-3)

Gal-3, a member of the carbohydrate-binding lectin family, plays a pivotal role in numerous pathophysiological processes, including the exacerbation of systemic inflammation and tissue fibrosis. Clinically, its serum levels demonstrate an inverse relationship with the ABI. A long-term prospective study demonstrated that serum Gal-3 concentrations were not significantly associated with all-cause mortality in patients presenting with PAD in the absence of CLTI, yielding a HR of 1.17 (CI: 0.96–1.42) over a 9.2-year follow-up period. Conversely, urinary Gal-3 excretion exhibits a robust correlation with long-term all-cause mortality (HR: 1.60; 1.31–1.95), remaining a strong independent predictor even after comprehensive adjustment for renal function parameters and traditional cardiovascular risk factors [40,41].

### 3.7. Growth Differentiation Factor 15 (GDF-15)

GDF-15 is a cytokine and an important biomarker for predicting clinical outcomes in PAD. Li B. et al. demonstrated in a prospectively enrolled cohort of 454 patients diagnosed with PAD that utilizing explainable statistical and machine learning methodologies, which included plasma GDF-15 levels alongside demographic and clinical characteristics, yielded exceptional predictive performance for 2-year MALEs in PAD patients: AUROC 0.84, accuracy 83.5%, sensitivity 83.6%, specificity 83.7%, positive predictive value (PPV) 87.3%, and negative predictive value (NPV) 86.2%. Furthermore, the serum GDF-15 levels increase with advancing Fontaine class. Kaplan–Meier analysis shows that the high-GDF15 group (≥2275 pg/mL) has higher all-cause mortality and increased thrombotic and hemorrhagic events compared to the low-GDF-15 group (<2275 pg/mL). Multivariate Cox regression confirms that GDF-15 is an independent predictor of all-cause mortality and such events, even after adjusting for confounding factors [42,43].

### 3.8. Fatty Acid-Binding Proteins (FABPs)

FABPs represent a family of intracellular chaperones that facilitate the transport of hydrophobic ligands and play a critical role in lipid metabolism. Among these, FABP4—predominantly produced by adipocytes and macrophages—promotes the progression of atherosclerosis by interacting with signaling pathways associated with vascular inflammation, insulin resistance, and heightened cardiovascular risk. High FABP4 levels are strong predictors of severe limb complications, particularly in individuals with PAD. Research indicates that increased FABP4 levels elevate the risk of MALEs, requiring vascular interventions or amputations. Specifically, a significant association was found with 3-year MALEs and worsening PAD status, with adjusted hazard ratios of 1.18 and 1.17, respectively, both statistically significant [44].

### 3.9. High-Mobility Group Box-1 (HMGB-1)

HMGB-1, a non-histone nuclear protein, regulates gene expression and orchestrates the systemic inflammatory cascade response following vascular injury. In diabetic patients presenting with CLTI, elevated baseline levels of HMGB-1 are robustly associated with adverse clinical outcomes and major cardiovascular events following peripheral revascularization procedures. A study involving 201 diabetic patients with PAD and CLTI demonstrated that elevated levels of HGMB-1 in blood correlate with increased limb-related events (AUC = 0.75) and major cardiovascular events (AUC = 0.78) following revascularization [24,45].

### 3.10. Osteoprotegerin (OPG)

OPG is recognized as a key inhibitor of vascular calcification and is deeply integrated into the pathophysiological processes of atherosclerosis and chronic inflammation. In patients with PAD, increased serum levels of OPG serve as a reliable biomarker for both the presence and progression of the condition, demonstrating markedly elevated concentrations compared to healthy controls. Within high-risk populations—particularly diabetic cohorts presenting with CLTI undergoing peripheral revascularization—elevated baseline OPG levels are predictive of adverse vascular outcomes, correlating with a heightened incidence of MACEs and MALEs. However, the independent predictive value of OPG for MACEs remains a subject of ongoing debate; while elevated concentrations are consistently documented in PAD patients experiencing cardiovascular events, its status as an independent risk predictor often diminishes after adjusting for confounding clinical variables. This highlights the necessity for further robust research to fully clarify its distinct prognostic significance [24,26,46,47].

In Table 1, a summary of classical inflammatory biomarkers with prognostic value in PAD is presented.

**Table 1:** Summary of classical inflammatory biomarkers with prognostic value in PAD. ALE—acute limb event; AUC—area under curve; CKD—chronic kidney disease; CRP—C-Reactive Protein; CLTI—Chronic limb-threatening ischemia; FABPs—Fatty acid-binding proteins; EVT—Endovascular therapy; GDF-15—Growth Differentiation Factor 15; HMGB-1—High-Mobility Group Box-1; hs-CRP—high-sensitivity C-reactive protein; HR—hazard ratio; IC—intermittent claudication; IL-1—interleukin 1; IL-6—interleukin 6; IL-27—interleukin 27; LER—lower extremity revascularization; MACEs—major adverse cardiovascular events; MALEs—major adverse limb events; MPO—myeloperoxidase; OPG—Osteoprotegerin; PAD—peripheral artery disease; RCT—randomized controlled trial; TNF-α—Tumor Necrosis Factor-α.

Marker(s)	Study Design	Population	Sample Size (N)	Objective	Outcomes
CRP [22]	A systematic review and meta-analysis	PAD patients undergoing lower limb revascularization	1460	The association between the baseline CRP levels and postprocedural ALE	High baseline levels are predictive of ALE (target vessel revascularization, amputation, restenosis, disease progression, composite endpoint of any of these ALE) (HR, 1.09; 95% confidence interval, 1.00–1.18; *p* = 0.04)
hs-CRP [23]	Cohort retrospective, single center	PAD patients undergoing EVT for femoropopliteal occlusive disease	71	The association between preprocedural hs-CRP and MACEs and MALEs	Elevated hs-CRP values were associated with increased MALEs (HR, 4.015; 95% CI, 1.628–10.551; *p* = 0.003)
IL-1, IL-6, CRP, TNF-alpha, HMGB-1, OPG [24]	Cohort prospective, single center	Diabetic CLTI patients undergoing LER	264	The association between a panel of biomarkers and MACEs and MALEs	The biomarker panel significantly improved the prediction of incident events for MACEs (AUC = 0.98; 0.95, 0.99, *p* < 0.01) and MALEs (AUC = 0.94; 0.91, 0.98, *p* < 0.01).
hs-CRP, fibrinogen [27]	A systematic review and meta-analysis	PAD patients	21,473	The association between plasma biomarkers and cardiovascular events and mortality	Increased hs-CRP levels had a relative risk of 1.86 (1.48–2.33) for major adverse cardiovascular events and a relative risk of 3.49 (2.35–5.19) for mortality Increased fibrinogen was associated with an increased relative risk of mortality of 2.08 (1.46–2.97)
TNF-α, IL-1, IL-6, hs-CRP [26]	A systematic review and meta-analysis	PAD patients	4673	The association of specific inflammatory biomarkers with morbidity and mortality	Elevated levels are consistently linked to major risks—including loss of vascular patency, MACEs/MALEs and all-cause mortality
hs-CRP [29]	Cohort prospective, single center	PAD patients with IC	335	The associations between hs-CRP and clinical outcomes	Elevated levels were significantly associated with cardiovascular-related and malignancy-related deaths even after adjusting for other risk factors (hazard ratio 2.79; 95% confidence interval 1.66–7.17, *p* = 0.024)
IL-27 [35]	Cohort prospective, single center	PAD patients undergoing EVT	489	The association between preprocedural IL-27 and MACEs and MALEs	Elevated levels were an independent predictor of MACEs (HR 2.95; *p* = 0.039), but not MALEs
NLR and TNF-α [37]	A phase III RCT (GHAS trial)	CTLI patients	35	An investigation of gene expression and plasma biomarkers CLTI	Markers of mortality in CLTI
MPO [38]	Cohort prospective, single center	PAD patients with IC	110	A measure of biomarkers of neutrophil activation	High levels are a predictor of 6-month MACEs and/or MALEs (AUC = 0.74, 0.56–0.91, and a sensitivity and specificity of 0.80 and 0.65) for a cut-off of 108.37 ng/mL
MPO [39]	Cohort prospective, single center	PAD patients undergoing femoral artery endarterectomy	37	The histology of atheromatous plaques in the femoral artery and their association with subsequent cardiovascular events	A higher immunologic score has a significantly higher cumulative risk of MACEs (*p* = 0.014)
Gal-3 [41]	Cohort prospective, single center	PAD patients without CTLI and mild to moderate CKD	577	The association between serum and urinary Gal-3 and long-term survival	Elevated urinary Gal-3 is associated with increased mortality (HR 1.60; 1.31–1.95)
GDF-15 [42]	Cohort prospective, single center	PAD patients	454	To use explainable statistical and machine learning methods to assess the prognostic value of GDF15 for limb outcomes	Plasma GDF15 levels have important prognostic value for 2-year MALEs (AUC = 0.84, accuracy 83.5%, sensitivity 83.6%, specificity 83.7%, PPV 87.3%, and NPV 86.2%)
GDF-15 [43]	Cohort prospective, single center	PAD patients before initial EVT	200	The association between GDF-15 levels and all-cause mortality rate	In multivariate Cox proportional-hazards regression analysis, serum levels are associated with all-cause mortality and thrombotic and bleeding events (HR, 2.50; 1.67–3.73; *p* < 0.0001; HR, 2.30; 1.43–6.17; *p* < 0.0037)
FABP4 [44]	Cohort prospective case–control	PAD patients/without PAD patients	568 with PAD 279 without PAD	The prognostic ability of FABP4 in predicting PAD-related adverse limb events	Higher levels were significantly associated with 3-year MALEs (composite of vascular intervention or major amputation) (unadjusted HR, 1.19; 1.04–1.27; adjusted HR, 1.18; 1.03–1.27; *p* = 0.022) and worsening PAD status (unadjusted HR, 1.18; 1.13–1.31; adjusted HR, 1.17; 1.12–1.28; *p* < 0.001).
HMGB-1 [45]	Cohort prospective case–control	PAD patients and CLTI after LER	201	The association between serum HMGB-1 levels and MACEs and MALEs	Increased serum levels are associated with the incidence of MACEs (AUC = 0.78) and MALEs (AUC = 0.75)

## 4. Modern Inflammatory Biomarkers

### 4.1. The Neutrophil-to-Lymphocyte Ratio (NLR)

The NLR is a cost-effective and readily accessible biomarker derived from a standard complete blood count. It reflects the dynamic balance and complex interplay between the innate and adaptive immune systems. In patients diagnosed with PAD, the NLR serves as a robust, independent prognostic marker for long-term clinical outcomes. Specifically, a comprehensive meta-analysis encompassing this population revealed significant associations between an elevated baseline NLR and an increased incidence of adverse outcomes, including all-cause mortality, MACEs and MALEs. Within these pooled evaluations, NLR demonstrated strong predictive performance for the stated clinical endpoints, yielding an AUC that consistently exceeds 0.70, while an elevated ratio was associated with a doubling of risk for these adverse events. Furthermore, in advanced stages of PAD characterized by severe ischemia (ABI < 0.5), NLR (AUC = 0.682, *p* = 0.010) and PLR (AUC = 0.692, *p* = 0.006) have been identified as critical predictors linked to an elevated risk of subsequent limb amputation. The prognostic utility of this biomarker is equally valuable across different revascularization strategies for PAD. For patients undergoing endovascular interventions, a retrospective study utilizing ROC curve analysis identified pre-procedural NLR as a strong predictor of post-percutaneous transluminal angioplasty (PTA) mortality, yielding a robust AUC of 0.842 (*p* < 0.001) with an optimal prognostic cutoff value of 4.64. Similarly, in the context of open vascular surgery, a retrospective study by Cosarca et al. comprising 203 PAD patients with concomitant diabetes mellitus undergoing either open surgical or endovascular revascularization determined that a specific NLR cutoff value of 3.485 predicted major amputation with a sensitivity of 60.42% and a specificity of 72.44%. These findings align with broader retrospective data indicating that a high preoperative NLR can independently predict both a prolonged hospital length of stay and heightened short-term mortality in patients undergoing open lower extremity revascularization procedures [2,40,48,49,50]. More specifically, a preoperative NLR threshold > 4 (AUC: 0.698; sensitivity: 64.0%; specificity: 75.7%) is significantly associated with an increased incidence of 5-year MALEs and overall mortality in patients presenting with symptomatic common femoral artery (CFA) occlusive disease who underwent femoral endarterectomy. Regarding the surgical treatment of PAD, according to the findings of Russu et al., a preoperative NLR value > 3.95 was strongly associated with a 12-month primary patency failure rate of 16.47% compared to a success rate of 89.21% observed in the low-NLR cohort (*p* < 0.0001). Furthermore, this threshold predicted a significantly higher incidence of major amputations (42.35% vs. 2.88%; *p* < 0.0001) and an increased risk of overall mortality (27.06% vs. 2.88%; *p* < 0.0001) [51,52].

On the other hand, postoperative NLR serves as an independent predictor of late restenosis in patients undergoing DCB angioplasty for femoropopliteal arterial disease (OR: 1.404, 1.073–1.839). A postoperative NLR cutoff value ≥ 2.78 demonstrates a sensitivity of 80.8% and a specificity of 53.2% (AUC: 0.666, 0.541–0.791), effectively stratifying patients, as evidenced by a significantly higher incidence of late restenosis in the high-NLR cohort (32.3% vs. 9.1%; *p* = 0.002). Furthermore, a recent study demonstrates that combining rotational atherectomy with a drug-coated balloon leads to significantly lower postprocedural NLR values compared to the balloon-only approach, highlighting the utility of this ratio as both a prognostic biomarker and a dynamic indicator of treatment efficacy in peripheral arterial interventions. Conversely, a retrospective single-center investigation revealed no significant relationships between preoperative NLR, PLR, and restenosis (all *p* > 0.05). In multivariate logistic regression analysis, NLR was identified as the sole independent risk factor for death (OR: 6.91, 3.18–14.99, *p* = 0.001). Interestingly, a subgroup analysis from the meta-analysis by Huang et al. revealed that preoperative NLR assessment possessed superior prognostic value in predicting both long-term mortality and target vessel restenosis compared to postoperative testing [48,53,54,55,56].

An elevated NLR represents a major risk factor for major amputation and is strongly associated with a significantly reduced probability of AFS. González-Fajardo et al. analyzed data from 561 patients with CLTI who underwent elective revascularization, revealing a median follow-up of 31 months. A high pre-procedural NLR over 5.0 correlated with increased coronary artery disease and congestive heart failure. This cohort also showed a higher prevalence of serious illness (Rutherford Category 5) and reduced AFS (HR: 2.325, 1.732–3.121) in multivariate analysis. Moreover, higher baseline NLR levels are significantly associated with an increased risk of long-term mortality and major amputations, as well as a prolonged mean hospital length of stay. Su et al. studied 195 patients with CLTI classified as 4 or higher on the Rutherford Classification, who underwent PTA. Patients were divided into high (≥8) and low NLR groups based on ROC analysis. The elevated NLR group showed higher one-year all-cause mortality, cardiac-related mortality, major MACEs and MALEs. Multivariate analyses confirmed significant correlations for all-cause mortality (HR: 3.599, *p* < 0.001) and cardiac-related mortality (HR: 5.286, *p* < 0.001), though MACEs and MALEs were not significant. Also, in a cohort of 488 patients undergoing percutaneous lower extremity revascularization, 78.5% of whom presented with CTLI, an elevated NLR was associated with decreased AFS, diminished freedom from major amputation, and reduced survival over a four-year duration. Furthermore, Erdoğan et al. studied 268 patients with CLTI who were not candidates for revascularization and received effective medical therapy. They defined “non-response” (disease progression) as the main endpoint. ROC analysis identified thresholds for NLR and PLR. Multivariate analyses showed that an NLR ≥ 4.63 (HR: 3.983, *p* < 0.001) and PLR ≥ 151.24 (HR: 2.254, *p* = 0.016) were associated with non-responsiveness to therapy [57,58,59,60,61,62].

In patients with lower limb revascularization for Rutherford IIa or IIb acute limb ischemia, pre-operative NLR serves as a powerful independent predictor of 30-day death or amputation (OR: 1.28 per unit increase, 1.12–1.47), presenting excellent discriminative capacity (AUC: 0.86, 0.82–0.90). A validated NLR cutoff level ≥ 5.4 demonstrates a 90.5% sensitivity and 73.6% specificity for these acute outcomes. Furthermore, patients with a pre-operative NLR ≥ 5.4 exhibit significantly lower AFS rates at 30 days, 6 months, and 1 year. Additionally, a meta-analysis encompassing seven studies with a total of 1758 patients demonstrated that an elevated NLR was significantly associated with a three-fold higher risk of 30-day mortality or major amputation (OR: 3.05; 95% CI: 1.69–5.52; *p* < 0.001) [63,64].

### 4.2. The Platelet-to-Lymphocyte Ratio (PLR)

The PLR serves as a major indicator of both anatomical severity and clinical prognosis in patients with PAD, while also reflecting treatment efficiency. Specifically, baseline PLR correlates directly with the burden of stenotic lesions (*p* = 0.066) and acts as an independent predictor of limb loss in severe ischemic states characterized by an ABI < 0.5. Ultimately, this biomarker demonstrates a significant predictive accuracy for major amputation, yielding an AUC of 0.692 (*p* = 0.006). In patients with femoropopliteal disease undergoing surgical revascularization, an elevated preoperative PLR determined at hospital admission serves as a powerful, independent predictor of adverse clinical outcomes. Utilizing an optimal prognostic cutoff value of 142.13 (yielding 79.1% sensitivity and 82.6% specificity), a high baseline PLR strongly predicts 12-month primary patency failure, as well as significantly higher rates of major amputation and overall mortality during follow-up, except for specific advanced sub-cohorts such as Rutherford category 5. Also, following peripheral revascularization for lower extremity artery disease, either surgical or interventional, a significantly higher baseline PLR was observed in patients who subsequently required amputation compared to those who did not (170.2; 95% CI: 104.00–226.50 vs. 110.9; 95% CI: 87.31–162.30; *p* < 0.01), highlighting its predictive value for limb loss. After controlling for associated risk factors, PLR ≥ 151.24 (*p* = 0.016) was independently associated with no response to treatment. In a single-center retrospective study evaluating patients who underwent PTA for femoropopliteal artery (FPA) disease, the long-term mortality rate was significantly higher in the high-PLR cohort (*p* = 0.001). Conversely, no significant correlations were observed between baseline PLR levels and the incidence of target vessel restenosis (*p* > 0.05) [19,48,49,51,57].

A retrospective study demonstrates that routine admission biomarkers provide crucial prognostic insight for patients treated for CLTI. Both higher NLR and PLR levels directly impact hospital resource management, significantly predicting a prolonged length of stay (*p* = 0.001 and *p* = 0.002, respectively), which fully justifies their implementation as cost-effective screening tools in advanced peripheral arterial interventions [58].

The prognostic value of the PLR stems from its unique capacity to reflect the intricate balance between detrimental vascular inflammation and host immune competence. Platelets actively drive atheroprogression and vascular inflammation by releasing pro-inflammatory mediators, promoting leukocyte recruitment, and facilitating microthrombi formation on disrupted atherosclerotic plaques. Conversely, a diminished lymphocyte count reflects accelerated cellular apoptosis and a compromised immune response, which typically occur downstream of sustained, chronic systemic inflammation [19,65].

### 4.3. Lymphocyte-to-Monocyte Ratio (LMR)

The LMR represents an essential prognostic marker for PAD, exhibiting a strong negative correlation with inflammatory indices like NLR and PLR. Disease advancement is characterized by a profound reduction in LMR, which directly predisposes patients to poor clinical outcomes. Preoperative lymphopenia and monocytosis, resulting in a low LMR, are strongly linked to elevated major amputation rates post-revascularization, especially in the presence of concomitant diabetes. Statistically, an LMR cutoff of 2.55 was validated to predict the requirement for amputation. Additionally, LMR values below 3.1 serve as a critical alert for the presence of CLTI and heightened short-term mortality. Beyond hard clinical endpoints, a diminished baseline LMR is a significant predictor of healthcare resource utilization, correlating with a markedly prolonged hospital stay after PAD treatment [19,58].

### 4.4. The Systemic Immune-Inflammation Index (SII)

The SII, an inflammatory biomarker derived from standard complete blood counts, is calculated using the formula: platelet count × neutrophil count/lymphocyte count. This metric effectively reflects the intricate interplay between detrimental vascular inflammation, pro-thrombotic states, and host immune competence. Consequently, elevated baseline SII levels serve as a robust prognostic indicator of clinical outcomes in PAD, particularly in individuals undergoing endovascular interventions. Investigating this specific utility, a retrospective single-center study evaluated PAD patients who underwent PTA for femoropopliteal artery lesions. The primary findings indicated that the long-term mortality rate was significantly higher in the cohort with elevated pre-procedural SII levels. Furthermore, through ROC curve analysis, an optimal prognostic SII cutoff value > 800 was established as an independent and robust predictor of overall mortality in this high-risk population. Currently, the evidence regarding the capability of the SII to predict target vessel restenosis remains inconsistent. While one study demonstrated a significant association between elevated SII levels and restenosis in patients with lower extremity arteriosclerosis obliterans, alternative data failed to establish a robust correlation with mid-term restenosis rates. Although the SII frequently emerges as a significant predictor in univariate analyses, its independent prognostic power often diminishes when evaluated alongside other composite hematological markers. For instance, a comparative study focusing on post-endovascular mortality revealed that despite the SII exhibiting robust predictive accuracy (AUC = 0.67), it was ultimately outperformed by the Pan-Immune Inflammation Value (PIV) (AUC = 0.72), which demonstrated the highest overall discriminative performance [48,66,67].

### 4.5. The Systemic Inflammatory Response Index (SIRI)

The SIRI is a composite inflammatory biomarker capturing host immune-inflammatory status, calculated by multiplying the number of neutrophils and monocytes and dividing by the number of lymphocytes. Elevated baseline SIRI levels correlate with increased mortality in PAD patients undergoing endovascular interventions. In a cohort undergoing PTA for femoropopliteal disease, an optimal SIRI cutoff of 3.51 was identified as a strong predictor of post-procedural mortality (AUC = 0.831). Despite its strong association with survival outcomes, SIRI does not significantly correlate with mid-term target vessel restenosis. Methodologically, while SIRI significantly identifies non-survivors in univariate analysis, it frequently loses independent prognostic significance in multivariate models where NLR remains the only independent risk factor for death. In broad comparative marker assessments, SIRI demonstrated the lowest predictive accuracy (AUC = 0.58), whereas PIV achieved the highest (AUC = 0.72). Nevertheless, SIRI has proven highly sensitive in specific regenerative and acute scenarios, serving as an excellent indicator of wound healing in CLTI patients’ post-endovascular therapy, while both postoperative SIRI values and its delta dynamic represent significant predictors of in-hospital mortality following elective major vascular surgery for aortoiliac occlusive disease [48,66,67,68,69].

### 4.6. The Aggregate Index of Systemic Inflammation (AISI)

The AISI, calculated using the formula: (neutrophils x platelets x monocytes)/lymphocytes, is a novel composite biomarker with substantial prognostic utility in advanced PAD. Accumulating evidence highlights its value in predicting target vessel restenosis following endovascular interventions for superficial femoral artery (SFA) disease. Specifically, patients presenting with an elevated baseline AISI demonstrate a 2.13-fold increased risk of restenosis (HR: 2.133; 1.508–3.017; *p* < 0.001) alongside a significantly shorter median time to restenosis (6 months vs. 12 months in the low-AISI cohort). Beyond endovascular applications, preoperative AISI serves as a robust predictor of short-term surgical outcomes in patients undergoing elective major arterial revascularization. Multivariate evaluations demonstrate that among various inflammatory indices, AISI represents the sole independent risk factor for forecasting in-hospital mortality. In patients with Leriche syndrome undergoing major open revascularization surgery, baseline AISI levels were significantly higher in those who subsequently developed restenosis compared with those who maintained vessel patency. Furthermore, AISI levels exhibit a sharp, statistically significant rise from the preoperative baseline to the immediate postoperative phase, effectively capturing the intense physiological and inflammatory stress associated with major vascular reconstruction [66,68,70].

### 4.7. C-Reactive Protein-to-Albumin Ratio (CAR)

The CAR represents a novel composite biomarker that pairs an acute-phase inflammatory reactant (CRP) with a nutritional and anti-inflammatory marker (albumin). It serves as a valuable prognostic tool for predicting clinical outcomes in patients diagnosed with PAD, particularly those undergoing EVT. Meta-analytic data indicate that an elevated pre-procedural CAR is associated with a more than two-fold increase in mortality risk, demonstrating an AUC of 0.75 for predicting death. Conversely, a low pre-procedural CAR score yields a highly favorable NPV for survival, translating into a 90.5% probability of remaining event-free. Higher baseline CAR levels are significantly linked to a higher incidence of major MACEs, target vessel restenosis, and an accelerated risk of limb loss post-EVT; notably, for major amputations, the metric exhibits an AUC of 0.85 coupled with an impressive NPV of 97.8% [71,72,73,74].

### 4.8. Hemoglobin, Albumin, Lymphocyte, and Platelet (HALP) Score

The HALP score represents a novel prognostic index that simultaneously evaluates a patient’s systemic inflammatory and nutritional status, thereby refining risk stratification in lower extremity PAD. This metric demonstrates a robust negative correlation with PAD severity, wherein lower HALP scores consistently indicate more advanced clinical and anatomical stages. Specifically, research utilizing the TransAtlantic Inter-Society Consensus (TASC) II angiographic criteria has revealed that patients with severe PAD exhibit significantly elevated platelet counts concurrently with decreased hemoglobin and serum albumin concentrations, directly contributing to a depleted HALP score. A validated ideal cutoff value of 3.14 demonstrates exceptional predictive accuracy, yielding an AUC of 0.889 and an 81% probability of correctly identifying severe PAD presentations [75,76].

Table 2 presents a summary of modern inflammatory biomarkers with prognostic value in PAD.

## 5. Current Anti-Inflammatory Therapy in PAD

Atherosclerosis is a long-term immune-inflammatory disease, and one of the main areas of research for treatment is the management of “residual inflammatory risk” in PAD. At present, there are no large-scale anti-inflammatory drugs specifically approved for PAD; however, various targeted agents and conventional medications with anti-inflammatory properties have shown considerable promise in reducing inflammation and improving patient outcomes in clinical trials. Table 3 presents a comparative analysis of pharmacological and investigational agents in peripheral artery disease: clinical design, safety, cost, and guideline readiness.

**A. Targeted Anti-Inflammatory Agents**

### 5.1. Canakinumab

Canakinumab, a monoclonal antibody targeting IL-1β, has shown promise in cardiovascular risk reduction, as evidenced by the CANTOS trial (Canakinumab Anti-inflammatory Thrombosis Outcomes Study), which reported a 15% decrease in MACEs in post-MI patients with high CRP levels. A pilot study with 38 patients suffering from PAD indicated that canakinumab reduced IL-6 and CRP levels, resulting in improvements in both maximum and pain-free walking distances versus placebo. Despite these findings supporting the notion of anti-inflammatory atheroprotection, the use of canakinumab for PAD remains investigational and not yet validated as an established therapy. The current body of evidence is limited, particularly given the small sample size and the premature termination of the exploratory study due to insufficient efficacy. Furthermore, safety concerns have emerged, including increased rates of neutropenia and a higher risk of fatal infections or sepsis linked to treatment. Additionally, the high lifetime costs of approximately $457,982 per patient pose significant barriers to practical implementation, with analyses suggesting a 0% probability of cost-effectiveness unless costs are substantially reduced by 91% for viability in routine clinical practice [77,78].

### 5.2. Colchicine

Colchicine is an anti-inflammatory medication that has proven effective in reducing cardiovascular events in CAD. Based on the results of randomized controlled trials like COLCOT (Colchicine Cardiovascular Outcomes Trial) and LoDoCo2 (Low-Dose Colchicine 2 Trial), researchers propose that colchicine may help stabilize limb perfusion, decrease the necessity for repeated revascularizations, and prevent amputations, given that PAD and CLTI share similar atherosclerotic and inflammatory mechanisms as CAD [79,80,81,82,83]. However, due to the pathophysiological differences between PAD and CAD, coronary outcomes may not be directly translatable. PAD is characterized by a greater burden of atherosclerosis and inflammation, a higher propensity for thrombosis and peripheral embolization, and significant microcirculatory involvement. ⁠The current evidence regarding colchicine in PAD remains conflicting. A recent retrospective study involving patients with PAD treated with colchicine indicated a reduced risk of MALEs and cardiovascular mortality in comparison to a control group. Conversely, an “emulated trial” that used Medicare data found no evidence that colchicine reduced MACEs, MALEs, or death at two years, although critics argue this study was underpowered and had an insufficient follow-up period. Furthermore, colchicine’s effectiveness was not demonstrated in recent large trials for cerebrovascular disease (CONVINCE—Colchicine for Vascular Inflammation in Cerebrovascular Event Study; CHANCE3—Clopidogrel in High-risk patients with Acute Nondisabling Cerebrovascular Events 3 Trial) and one for acute myocardial infarction (AMI) (CLEAR-SYNERGY—Colchicine and Spironolactone Early in Acute Myocardial Infarction Revealing Synergy Trial), contributing to clinical uncertainty. Given these mixed findings, it must be explicitly stated that the use of colchicine for treating PAD is currently an investigational approach and not an established therapy. Prescribing colchicine for PAD lacks current guideline support and requires definitive validation through rigorous randomized controlled trials (RCTs) to ensure clinical efficacy and avoid compromising patient safety. To address this critical evidence gap, the LEADER-PAD (Low Dose Colchicine in Patients with Peripheral Artery Disease to Address Residual Vascular Risk) (NCT04774159) experiment is currently in progress. This randomized, double-blind, multi-center trial is examining the efficacy of low-dose colchicine in decelerating the course of PAD, preventing the failure of lower limb revascularization, and markedly decreasing the occurrence of MACEs and MALEs in this high-risk cohort [84,85,86,87,88].

### 5.3. Ziltivekimab

Ziltivekimab is a novel monoclonal antibody initially tested for safety and effectiveness in patients with rheumatoid arthritis and chronic kidney disease. Evidence suggests it may reduce the risk of CVD in individuals undergoing hemodialysis by suppressing inflammation. The large-scale ZEUS (Ziltivekimab Cardiovascular Outcomes Trial) trial (NCT05021835) is currently investigating the effect of ziltivekimab on cardiovascular outcomes in patients with established atherosclerotic CVD. If successful, this study could lead to a specific immunomodulatory treatment for peripheral artery disease [89,90].

**B. Traditional Therapies with Anti-Inflammatory Effects**

### 5.4. Statins

In contrast to novel immunomodulatory agents that currently remain investigational or lack consensus, statin therapy must be emphasized as the only therapeutic strategy targeting vascular inflammation that is fully supported by robust, PAD-specific clinical evidence and formal guideline implementation. Beyond their primary lipid-lowering effects, the extensive clinical benefits of statins in reducing MACEs and MALEs are inherently driven by their systemic pleiotropic anti-inflammatory properties. This pivotal role is definitively underscored by the 2024 ESC Guidelines for the Management of Peripheral Arterial and Aortic Diseases and 2024 ACC/AHA Guideline for the Management of Lower Extremity Peripheral Artery Disease, which assign a Class I, Level of Evidence A recommendation to long-term high-intensity statin therapy for all PAD patients. Clinical data and meta-analyses suggest that patients with PAD receiving statin therapy exhibit a 38–39% lower risk of all-cause mortality and a 41% reduction in cardiovascular mortality. Furthermore, statins can decrease MACEs, such as MI and stroke, by up to 50%. Statin medication also significantly mitigates the risk of MALEs, lowering amputation rates by 25–35% and increasing amputation-free survival by up to 56%. Beyond survival benefits, statins improve walking capacity, alleviate symptoms of IC, and delay the functional decline of the lower extremities. Interestingly, recent studies indicate that the clinical efficacy of statins in PAD patients is modulated by their baseline inflammatory status. Observational data reveal that statins are highly effective in reducing adverse outcomes, including death and major amputation, particularly in patients with elevated CRP levels (>1.0 mg/dL). Conversely, statins do not demonstrate a statistically significant independent predictive benefit in PAD patients with low-to-moderate baseline CRP levels. These findings underscore that a primary mechanism driving the benefit of statins in advanced PAD is their capacity to suppress active, high-grade vascular inflammation [5,82,91,92,93,94]. Consequently, while the therapeutic landscape of residual inflammatory risk continues to evolve, statins represent the absolute cornerstone of current guideline-directed medical therapy, translating anti-inflammatory efficacy into hard clinical outcomes in routine practice.

### 5.5. Proprotein Convertase Subtilisin/Kexin Type 9 Inhibitors (PCSK9i)

PCSK9i, including monoclonal antibodies such as evolocumab and alirocumab, represent a potent therapeutic approach for lipid reduction in individuals with PAD. Landmark randomized controlled trials have demonstrated that PCSK9 inhibitors significantly lowered the risk of MACEs and MALEs. In the FOURIER Trial (Further Cardiovascular Outcomes Research With PCSK9 Inhibition in Subjects With Elevated Risk), evolocumab significantly reduced the risk of cardiovascular mortality, MI, or stroke by 27% in patients with established PAD. Because patients with PAD carry a higher baseline cardiovascular risk, they experience a reduction in MACE risk (3.5%) compared to those without PAD (1.4%). Crucially, evolocumab also lowered the incidence of MALEs (ALI, major amputation, and urgent revascularization) by 37%. Furthermore, in patients with CLTI receiving evolocumab therapy alongside maximally tolerated statin, the drug was associated with a robust reduction in LDL-C, a decrease in carotid intima-media thickness (IMT), and an improvement in vascular flow-mediated dilation (FMD) [20,94,95,96,97]. Another study investigating the impact of evolocumab on limb outcomes in CLTI patients indicated that treatment correlated with improved amputation-free survival (AFS) and that long-term therapy (over 12 months) enhanced the rate of wound-free limb salvage. Moreover, adding evolocumab to maximally tolerated statin therapy improves maximum walking duration, increases FMD, and reduces IMT in patients with PAD and IC. Similarly, the ODYSSEY OUTCOMES trial (Evaluation of Cardiovascular Outcomes After an Acute Coronary Syndrome During Treatment With Alirocumab) demonstrated that alirocumab reduced the risk of PAD-specific outcomes (such as CLTI, limb revascularization, or amputation) by 31%. Interestingly, researchers noted that the reduction in these adverse limb events was specifically linked to the lowering of lipoprotein(a) [Lp(a)] levels, rather than being solely driven by the decrease in LDL cholesterol [20,98,99,100,101].

### 5.6. Inclisiran

Inclisiran, a small interfering RNA (siRNA) therapeutic, was recently approved for the treatment of familial hyperlipidemia following the successful ORION clinical trials (Outcomes Research with Inclisiran on Ischemic Outcomes in New or Established Cardiovascular Disease Patients). Despite its established protective role in ASCVD, further investigation is required to definitively clarify its impact on MACEs and MALEs in patients diagnosed with PAD. To address this evidence gap, the ongoing HPS-4/TIMI65 ORION-4 trial (NCT03705234) is currently evaluating the effects of inclisiran on major cardiovascular outcomes, including cardiovascular mortality, nonfatal MI, and urgent coronary revascularization. In parallel, the SIRIUS (Simulation of Inclisiran Readiness and Impact utilizing Unified Simulations) in silico modeling program recently assessed the efficacy of inclisiran on MACEs using a mechanistic simulation involving 204,691 virtual patients with ASCVD. Over a simulated 5-year period, comparing inclisiran to placebo as an adjunct to high-intensity statin therapy, the model predicted a 49.7% reduction in LDL-C. Furthermore, the simulation indicated a lower risk of 3-point MACEs, MI, ischemic stroke, and MALEs, alongside a predicted 7.1% relative reduction in cardiovascular death [102,103].

### 5.7. Ezetimibe

Ezetimibe is a lipid-lowering medication recommended for patients with PAD who fail to achieve target LDL-C levels despite dietary modifications and maximally tolerated statin therapy. A secondary analysis of the IMPROVE-IT trial (Improved Production of Outcomes: Vytorin Efficacy International Trial) demonstrated that combining ezetimibe with simvastatin significantly reduced major cardiovascular events in high-risk vascular patients, particularly those with polyvascular disease. Beyond lowering LDL-C by approximately 14 mg/dL, the clinical benefits of ezetimibe may be partially attributed to its anti-inflammatory and pleiotropic properties rather than its lipid-lowering actions alone. These ancillary mechanisms include the downregulation of pro-inflammatory cytokines and the preservation of endothelial function. Nevertheless, further research is warranted to fully elucidate the independent anti-inflammatory effects of ezetimibe monotherapy and to characterize how they compare with its combined use alongside statins [94,98,104].

### 5.8. Icosapent Ethyl (IPE)

Icosapent ethyl, an ethyl ester of eicosapentaenoic acid, significantly improved ischemic outcomes in the landmark REDUCE-IT trial (Reduction of Cardiovascular Events with Icosapent Ethyl International Trial). In this study, participants receiving 4 g/day of IPE as an adjunct to statin therapy experienced a 30% reduction in major cardiovascular events (HR = 0.70, *p* < 0.0001). Notably, this intervention demonstrated substantial clinical advantages regarding composite endpoints, specifically in the subgroup of patients with established PAD [105].

### 5.9. Bempedoic Acid

Bempedoic acid is a novel lipid-lowering agent evaluated in the landmark CLEAR Outcomes trial (Colesterol Lowering via Bempedoic Acid an EDC-Antagonist Randomized Outcomes Trial). The study demonstrated significant reductions in composite cardiovascular events among patients presenting with documented statin intolerance. However, further targeted research remains warranted to fully elucidate its efficacy and clinical outcomes specifically within cohorts with established PAD and related ischemic events [106,107].

### 5.10. Cilostazol

Cilostazol, a phosphodiesterase 3 (PDE3) inhibitor, is approved as a first-line medical therapy for IC. At a standard regimen of 100 mg twice daily, it significantly improves both initial and maximum walking distances compared to placebo. Clinical guidelines recommend its use primarily in patients severely limited by claudication who lack access to or are unable to participate in supervised exercise training programs. Therapy should be discontinued if a meaningful symptomatic improvement is not observed after three months. Beyond symptom relief, cilostazol effectively maintains arterial patency following surgical and endovascular interventions—particularly within the femoropopliteal segment—by suppressing intimal hyperplasia. Furthermore, incorporating cilostazol into standard antiplatelet therapy after EVT improves primary patency while lowering the rates of in-stent restenosis, target lesion revascularization, and arterial re-occlusion. Notably, the DORIC trial (Diabetic Peripheral Artery Disease Outcomes with Rivaroxaban and Intensive Cilostazol Treatment) demonstrated that adjunctive cilostazol reduced the risk of acute ischemic events, enhanced the ABI, and prolonged walking distances in diabetic patients with PAD concurrently taking clopidogrel, without increasing the risk of major bleeding complications. Additionally, current evidence indicates that cilostazol accelerates the healing process of diabetic foot ulcers significantly more effectively than aspirin monotherapy [108,109,110,111,112,113,114,115,116].

### 5.11. Antiplatelet Therapy

Antiplatelet therapies are crucial in the management of PAD, given their capacity to mitigate systemic atherosclerosis and atherothrombosis. Single antiplatelet therapy (SAPT) with either aspirin or clopidogrel constitutes the cornerstone of treatment for symptomatic PAD to lower the risk of MI, stroke, and vascular death. While aspirin lowers the risk of MACEs by 22%, it concurrently raises the risk of major bleeding complications by 60%, and robust evidence demonstrating its efficacy in preventing MALEs remains lacking. Data from the landmark CAPRIE trial (Clopidogrel versus Aspirin in Patients at Risk of Ischemic Events) support the use of clopidogrel, which demonstrated an 8.7% relative reduction in MACE risk compared to aspirin, alongside a more favorable tolerability profile. Furthermore, the EUCLID trial (Examining Use of tiCagreLor In paDisease) established that ticagrelor did not surpass clopidogrel in preventing either cardiovascular or limb-related adverse events [20,98,117].

Dual antiplatelet therapy (DAPT), consisting of aspirin combined with an inhibitor, is generally not recommended for patients with chronic PAD unless they present with a high atherothrombotic risk. Data from the CHARISMA study (Clopidogrel for High Atherothrombotic Risk and Ischemic Stabilization, Management, and Avoidance) demonstrated that the combination of aspirin and clopidogrel did not significantly reduce MACEs in symptomatic PAD patients, while significantly increasing the risk of bleeding complications. Nonetheless, short-term DAPT may be utilized following endovascular interventions. Conversely, the PEGASUS-TIMI 54 trial (Prevention of Cardiovascular Events in Patients With Prior Heart Attack Using Ticagrelor Against Placebo on a Substantial Undiscovered Subgroup–Thrombolysis In Myocardial Infarction 54) established that combining aspirin with ticagrelor significantly lowered MACEs in high-risk PAD patients with a prior history of MI, while concurrently reducing the incidence of MALEs by 40% compared to aspirin monotherapy [118,119].

A novel therapeutic approach for PAD utilizes dual pathway inhibition (DPI), combining antiplatelet therapy with low-dose anticoagulation, specifically aspirin and low-dose rivaroxaban (2.5 mg twice daily). The landmark COMPASS trial (Cardiovascular Outcomes for People Using Anticoagulation Strategies) demonstrated that this combination significantly reduced MACEs, MALEs, and major amputations. Furthermore, the VOYAGER PAD trial (Vascular Outcomes StudY of ASA alonG with Rivaroxaban in Endovascular or Surgical Limb Revascularization for PAD) revealed a 15% reduction in the composite risk of acute limb ischemia, major amputation, MI, and ischemic stroke following revascularization. Although DPI significantly elevates the risk of major bleeding complications, it does not increase the risk of fatal or intracranial hemorrhage, thereby demonstrating a favorable net clinical benefit-to-risk ratio. Conversely, the WAVE trial (Warfarin Antidote and Vascular Events Trial (Warfarin Antiplatelet Vascular Evaluation) showed that combining warfarin with antiplatelet therapy offered no cardiovascular or limb benefits, while resulting in an intolerable 3- to 4-fold excess risk of life-threatening bleeding. Additionally, the TRA2P-TIMI 50 trial (Thrombin Receptor Antagonist in 2ndary Prevention of Atherothrombotic Ischemic Events–Thrombolysis In Myocardial Infarction 50) indicated that adding vorapaxar, a protease-activated receptor-1 (PAR-1) antagonist, to standard antiplatelet therapy lowers the risk of MACEs and ALI. However, its clinical use is strongly advised against because it drastically increases the risk of severe bleeding, including cerebral hemorrhage, and is strictly contraindicated in patients with a history of stroke [20,96,120,121,122].

### 5.12. Sodium-Glucose Co-Transporter 2 Inhibitors (SGLT2Is)

SGLT2Is have been shown to attenuate atherosclerosis and promote vulnerable plaque stabilization, thereby exerting systemic cardiovascular benefits and reducing vascular inflammation, particularly in the context of PAD. Robust clinical evidence demonstrates that SGLT2Is reduce MACEs, heart failure hospitalizations, and cardiovascular mortality. Although initial findings from the CANVAS trial (Canagliflozin Cardiovascular Assessment Study) raised safety concerns regarding an increased risk of lower-limb amputations with canagliflozin, subsequent large-scale data successfully refuted this signal. Specifically, the landmark EMPA-REG OUTCOME (Empagliflozin Regulatory Outcome Trial) and DECLARE-TIMI 58 trials (Dapagliflozin Effect on Cardiovascular Events–Thrombolysis In Myocardial Infarction 58) established that SGLT2Is (empagliflozin and dapagliflozin, respectively) confer substantial cardiovascular protection in patients with PAD without escalating amputation rates. Furthermore, following statistical adjustments, SGLT2I exposure was correlated with a lower incidence of new-onset PAD and its associated adverse outcomes compared to dipeptidyl peptidase-4 inhibitors (DPP-4Is). Conversely, data from a separate retrospective cohort study suggested that incorporating SGLT2Is into antidiabetic regimens might elevate the likelihood of requiring PAD-related surgical interventions when compared to DPP-4I therapy [123,124,125,126,127,128,129].

### 5.13. Glucagon-like Peptide-1 (GLP-1) Receptor Agonists

GLP-1 receptor agonists significantly reduce the risk of ischemic events in patients with PAD, exerting protective effects that extend beyond glycemic control. Data from notable cardiovascular outcomes trials, such as LEADER (Liraglutide Effect and Action in Diabetes: Evaluation of Cardiovascular Outcome Results) and SUSTAIN (Semaglutide Unabated Sustainability in Treatment of Type 2 Diabetes), demonstrate that these medications lower MACEs and cardiovascular mortality, showing pronounced efficacy particularly in preventing ischemic events. For instance, liraglutide therapy is associated with an approximate 35% reduction in amputation risk among patients with PAD, underscoring the clinical benefits of early therapeutic initiation. Furthermore, GLP-1 receptor agonists have been shown to enhance vascular function, as evidenced by significant improvements in FMD early during treatment. To further explore these specific limb-related benefits, the ongoing STRIDE trial (Semaglutide Treatment on Walking Distance in Peripheral Artery Disease) is currently evaluating the impact of semaglutide on walking capacity and the ABI in this high-risk population [20,98,128].

### 5.14. Dipeptidyl Peptidase-4 Inhibitors (DPP4Is)

DPP-4Is are antidiabetic agents that, despite demonstrating some potential vascular benefits, exert a neutral effect on ischemic outcomes and fail to significantly reduce MACEs. Compared to SGLT2Is—which provide a 21% relative risk reduction for PAD—DPP-4Is exhibit limited efficacy in preventing ischemic events or slowing PAD progression. Nevertheless, they maintain a well-established safety profile in patients at high cardiovascular risk [20,89,123].

**Table 3:** Comparative analysis of pharmacological and investigational agents in peripheral artery disease: clinical design, safety, cost, and guideline readiness.

Therapeutic Agent	Primary Trial Design and Population	Level of Evidence for PAD	Major Safety Concerns	Cost and Accessibility Considerations	Clinical Readiness
Canakinumab (IL-1β Inhibitor)	CANTOS trial: Post-MI patients with high CRP [77]	ASCVD RCT with PAD subgroup	Increased rates of neutropenia Higher risk of fatal infections or sepsis [78]	Extremely High Cost: ~$457,982 lifetime cost/patient 0% probability of cost-effectiveness unless costs are cut by 91% [130]	Low/Investigational: Not validated or established as a standard routine therapy for PAD
	Pilot study: 38 PAD patients [77]	Small exploratory PAD study (terminated prematurely)			
Colchicine	COLCOT and LoDoCo2 trials: ASCVD patients [79,131]	ASCVD RCT with PAD subgroup	Gastrointestinal side effects Potential uncertainty highlighted by neutral data in other vascular trials (CONVINCE, CHANCE3 and CLEAR-SYNERGY) [84,86,88]	Low cost and high accessibility: widely available as a cheap generic medication [81]	Moderate / Investigational: Lacks current guideline support or definitive clinical validation for PAD Pending the results of the LEADER-PAD trial
	LEADER-PAD trial (Ongoing): Dedicated high-risk PAD cohort [87]	High-risk PAD patients			
Ziltivekimab (IL-6 Inhibitor)	ZEUS trial (Ongoing) Established ASCVD and PAD [132]	ASCVD RCT with PAD subgroup (Ongoing large-scale evaluation)	Mild injection site reactions Potential minor lipid changes Notable, beneficial increase in hemoglobin levels [90]	No commercial costs and strictly limited access to clinical trial participants: not yet approved by the FDA or EMA	Low/ Investigational Awaiting outcomes in PAD patients from the large-scale ZEUS trial
	RESCUE trial: High atherosclerotic risk [90]	CKD RCT on high-atherosclerotic-risk patients			
Statins	Multiple landmark cardiovascular prevention trials and large observational databases [5,93]	PAD-specific Meta-analyses and Large Observational Cohorts (Strong indirect/subgroup RCT evidence)	Myalgia/Myopathy Elevated liver enzymes (rare) [133]	Very low cost and ubiquitous accessibility: universally available generic drug [134]	Very high: first-line guideline-directed medical therapy for all PAD patients [5,93]
PCSK9 Inhibitors (Evolocumab /Alirocumab)	FOURIER (Evolocumab): Established ASCVD/PAD subgroup [97]	ASCVD RCT with prespecified PAD subgroups	Injection site reactions Nasopharyngitis [135]	High cost: represents a significant economic barrier compared to standard statins [136]	High: guideline-directed medical therapy for high-risk vascular patients, statin intolerance, or uncontrolled lipids [5,93]
	ODYSSEY OUTCOMES (Alirocumab): Post-ACS with PAD subgroup [137]				
Inclisiran (siRNA)	ORION trials [102]	Familial hyperlipidemia/ASCVD	Injection site reactions Preclinical/In silico modeling for PAD [102]	High cost: requires professional administration via a twice-yearly injection schedule [138]	Moderate: Approved for general lipid-lowering in ASCVD, but its specific readiness for PAD outcomes awaits clinical RCT evidence [5,93]
	ORION 4 trial (Ongoing)	ASCVD RCT and PAD patients			
Ezetimibe	IMPROVE-IT trial [139]	ASCVD RCT with PAD Subgroup	Generally well tolerated Rare mild gastrointestinal disturbances or myalgias (usually when combined with statins) [5,93]	Low cost and high accessibility: widely available as an affordable generic drug globally [140]	Very high: highly established second-line lipid-lowering therapy recommended by guidelines when statin targets are not met [5,93]
	Multiple observational cohorts and lipid guidelines [5,93]	Strong clinical database evidence			
Bempedoic Acid	CLEAR Outcomes trial; statin-intolerant patients with high cardiovascular risk or established ASCVD [106]	ASCVD RCT with PAD Subgroup	Hyperuricemia (increased risk of gout) Small risk of tendon rupture Elevated liver enzymes [5]	Moderate cost: more expensive than generic statins/ezetimibe [141]	High: guideline-recommended medical therapy as a potent alternative/adjunct for high-risk PAD patients with statin intolerance [5]
Icosapent Ethyl (IPE)	REDUCE-IT: Patients with established ASCVD or diabetes, plus elevated triglycerides despite statin therapy [105]	ASCVD RCT with prespecified PAD subgroup	Increased risk of atrial fibrillation/flutter Slight increase in minor bleeding risks [105]	Moderate-to-high cost: Subject to regional availability and specific prior-authorization policies based on triglyceride levels [142]	High: strongly endorsed by international guidelines for high-risk vascular patients with persistent hypertriglyceridemia [5,93]
Cilostazol (PDE3 Inhibitor) focusing on IC and post-EVT patency	Multiple dedicated PAD trials [5,93]	PAD-Specific RCTs	Headache, diarrhea, palpitations. Contraindicated in patients with heart failure.	Low-to-moderate cost: readily accessible and affordable [143]	Very low: current clinical 2024 ESC Guidelines do not recommend cilostazol for the explicit purpose of MACE reduction or in the context of LEAD management [5,93] Very High: Approved and universally designated as a first-line medical treatment for severe IC according to 2024 ACC/AHA Guidelines [93]
Dual Pathway Inhibition (Aspirin + Rivaroxaban)	COMPASS and VOYAGER PAD: Stable vascular disease and post-revascularization PAD patients [5,93]	PAD-Specific RCTs (Large-scale dedicated multi-center trials)	Significantly increased risk of major bleeding (though fatal/intracranial bleeding is not significantly increased) [5,93]	Moderate cost: Rivaroxaban (2.5 mg bid) requires specific healthcare insurance/prescription authorization depending on regional rules [144]	Moderate: should be considered for patients with PAD and non-high bleeding risk at high ischemic risk or following lower-limb revascularization [5] High: in symptomatic PAD patients with no recent revascularization/recent revascularization (endovascular or surgical) [93]
SGLT2 Inhibitors (Dapagliflozin/Empagliflozin)	EMPA-REG, DECLARE-TIMI 58: Type 2 Diabetes and ASCVD cohorts with PAD subgroups [125,127]	ASCVD RCT with PAD subgroup	Genital mycotic infections [5,93]	Moderate-to-high cost: highly accessible for metabolic/diabetic indications [145]	High: fully ready and increasingly used for diabetic patients with concurrent PAD due to established systemic cardioprotective benefits [5,93]
GLP-1 Receptor Agonists (Liraglutide/Semaglutide)	LEADER and SUSTAIN: Type 2 diabetes and high cardiovascular risk cohorts [128,146] STRIDE (Ongoing): Dedicated symptomatic PAD and type 2 diabetes trial [128]	ASCVD RCT with PAD Subgroup	Gastrointestinal side effects (nausea, vomiting) Risk of dehydration [147]	High cost: substantial economic consideration, although widely covered for diabetic indications [148]	High: fully ready and increasingly used for diabetic patients with concurrent PAD due to established systemic cardioprotective benefits [5,93]
DPP-4 Inhibitors (DPP4Is)	Multiple large cardiovascular safety trials in type 2 diabetes populations [89,123]	ASCVD RCT with PAD Subgroup	Generally safe and well tolerated Minimal gastrointestinal side effects [149]	Moderate cost: widely available and accessible for diabetic patients [150]	Low: Highly ready as safe antidiabetic drugs, but show a neutral effect on ischemic outcomes and fail to slow PAD progression They lack current guideline support for PAD management.

ACC—American College of Cardiology; AHA—American Heart Association; ASCVD—atherosclerotic cardiovascular disease; DPP-4—dipeptidyl peptidase-4; ESC—European Society of Cardiology; EMA—European Medicines Agency; EVT—Endovascular therapy; FDA—The U.S. Food and Drug Administration; GLP-1—glucagon-like peptide 1; IC—intermittent claudication; IL-1β—interleukin-1β; IL-6—interleukin 6; LEAD—Lower Extremity Arterial Disease; MACEs—major adverse cardiovascular events; MI—myocardial infarction; PAD—peripheral artery disease; PDE3—Phosphodiesterase 3; RCT—randomized controlled trial; SGLT2—Sodium-Glucose Cotransporter 2; siRNA—small interfering RNA.

## 6. Strengths, Limitations, and Confounding Factors in Current Evidence

Although current evidence underscores the prognostic value of inflammatory biomarkers in PAD, greater attention should be given to the characteristics of the included patient populations and factors that may affect generalizability. Many of the available findings are derived from retrospective, single-center studies with relatively small sample sizes. Furthermore, certain cohorts present demographic imbalances, such as a significant male predominance, meaning the results cannot be automatically generalized to the entire PAD population.

A major challenge in establishing the true prognostic value of biomarkers such as CRP, the NLR, and the PLR lies in the presence of important clinical confounders. Inflammatory markers are highly sensitive but lack disease specificity. For example, diabetes mellitus and CKD inherently generate a chronic, low-grade systemic inflammatory status. Diabetes accelerates atherosclerosis through hyperglycemia, insulin resistance, and oxidative stress, thereby intrinsically elevating pro-inflammatory cytokines. Similarly, CKD induces a pro-atherosclerotic and pro-calcific state accompanied by systemic inflammation. Consequently, in patients with multiple comorbidities, it is difficult to determine whether elevated biomarkers uniquely reflect the severity of limb ischemia or merely the overall inflammatory burden of these systemic conditions [31,36,45,46,151].

Infection and active malignancy are other critical confounders that can drastically skew biomarker levels. Patients with CLTI often present with severe tissue loss, infected foot ulcers, or underlying systemic infections, which artificially spike leukocyte counts and acute-phase reactants. While some rigorous studies explicitly exclude patients with elevated white blood cell counts, active infections, autoimmune diseases, or a recent history of malignancy to limit this bias, other studies do not account for these conditions. This inconsistency represents a critical limitation that may distort the relationship between biomarkers and vascular prognosis [36,60].

Concomitant medications also significantly affect the inflammatory profile. Most PAD patients are on optimal medical therapy, which includes statins, antiplatelet agents, and angiotensin-converting enzyme inhibitors—drugs well known for their pleiotropic anti-inflammatory properties. The lack of standardized data regarding the dosage, timing, and duration of these cardiovascular risk-reduction therapies, alongside the potential unrecognized use of immunosuppressive agents or systemic corticosteroids, often undermines the predictive power of biomarkers in multivariate analyses [46,70].

A more balanced discussion of the available evidence highlights both clear strengths and notable limitations. On the one hand, a major strength of markers like the NLR, PLR, and CRP is their widespread availability, cost-effectiveness, and ease of integration into routine clinical practice. On the other hand, the scientific rigor of the current literature is constrained by the lack of universally accepted cut-off values for these indices, leading to high heterogeneity across studies. Moreover, most studies rely on a single baseline measurement of these biomarkers, failing to track dynamic post-procedural variations that might better capture the ongoing intervention-induced inflammatory response or resolution of ischemia. Addressing these limitations through large-scale, prospective, multicenter studies that meticulously adjust for these confounders is essential to improve the clinical relevance and therapeutic applicability of inflammatory biomarkers in PAD [2,48,52,60,152].

## 7. Future Direction

While inflammatory biomarkers show significant promise in predicting the progression of PAD, extensive validation is required before their widespread translation into routine clinical settings. In particular, further investigations are critical to establish and standardize the exact cutoff values for indices such as the NLR and the CAR. Resolving these existing inconsistencies is paramount, as they currently preclude the formal integration of these biomarkers into international clinical guidelines. Additionally, prospective studies must elucidate the precise prognostic value of emerging composite markers, such as the SII and the SIRI, regarding restenosis rates and overall surgical outcomes. Rather than adhering to conventional, non-specific anti-atherosclerotic strategies, modern vascular medicine should pivot toward targeted precision medicine to systematically address the “residual inflammatory risk”. In this context, ongoing randomized clinical trials—including LEADER-PAD, ZEUS, and ORION-4—remain essential for rigorously evaluating the therapeutic efficacy of novel anti-inflammatory and intensive lipid-lowering interventions within high-risk cohorts. Furthermore, future clinical trial designs should systematically incorporate limb-specific endpoints, such as wound healing kinetics and amputation-free survival rates. Concurrently, advancements in machine learning and artificial intelligence present a revolutionary opportunity to develop multi-dimensional predictive models that dynamically integrate biomarkers, genomic profiles, and comprehensive clinical data. Ultimately, this holistic approach will facilitate the delivery of highly tailored, personalized therapeutic regimens for individuals diagnosed with peripheral artery disease.

## 8. Conclusions

In conclusion, PAD is recognized as a multifaceted systemic inflammatory disorder rather than merely a localized concern. Conventional risk factor management continues to expose patients to considerable inflammatory risks that may result in plaque instability and unfavorable outcomes. Using modern inflammatory biomarkers in clinical assessments, like the NLR and the CAR, can help identify patients who are at high risk for serious cardiovascular events. To improve survival, preserve limb function, and improve the quality of patient care, future management strategies should focus on treating vascular inflammation with new immunomodulatory drugs.

## Figures and Tables

**Table 2:** Summary of modern inflammatory biomarkers with prognostic value in PAD. ABI—ankle-brachial index; AISI—The Aggregate Index of Systemic Inflammation; ALI—acute limb ischemia; ASO—arteriosclerosis obliterans; CAR—C-reactive protein-to-albumin ratio; CLTI—Chronic limb-threatening ischemia; FPA—femoropopliteal artery; EVT—endovascular therapy; DCB—drug-coated balloon; HALP score—Hemoglobin, Albumin, Lymphocyte, and Platelet score; MALEs—major adverse limb events; NLR—Neutrophil-to-Lymphocyte Ratio; PAD—peripheral artery disease; PVI—Peripheral vascular intervention; PLR—Platelet-to-Lymphocyte Ratio; PTA—Percutaneous transluminal angioplasty; RC—Rutherford classification; SFA—Superficial femoral artery; SII—Systemic Immune-Inflammation Index; SIRI—Systemic Inflammatory Response Index.

Marker(s)	Study Design	Population	Sample Size (N)	Objective	Cut-off Value (ng/L)	AUC	Sensitivity (%)	Specificity (%)	Outcomes
NLR [48]	Cohort retrospective, single center	PAD patients undergoing PTA for FPA disease	418	The association between preoperative inflammation-related biomarkers and mid-term restenosis and mortality	>4.64	0.842	69.23	88.95	NLR was independently associated with mortality (HR: 6.91; 3.18–14.99, *p* = 0.001)
NLR PLR [49]	Cohort retrospective, single center	PAD patients	652	The diagnostic and prognostic role of inflammatory markers	-	0.682 0.692	-	-	High levels of NLR (CI: 0.419–0.664) and PLR (CI: 0.556–0.829) were predictors associated with a high risk of amputation in patients with an ABI < 0.5
NLR [50]	Cohort prospective, single center	PAD patients undergoing open lower extremity revascularizations	535	The association between NLR and mortality	>4.6	-	0.61	0.74	Preoperative NLR as both a dichotomous (NLR </> 4.6) variable(HR 2.78; 1.75–4.35, *p* < 0.0001) andas a continuous variable (HR 1.04; 1.02–1.06, *p* < 0.0004) was found to be an independentpredictor of mortality
NLR PLR [51]	Cohort retrospective, single center	PAD patients with femoropopliteal disease	224	The role of NLR and PLR in the medium-term outcome of patients surgically revascularized	>3.95 >142.13	-	82.6 79.1	89.9 82.6	High values of preoperative NLR and PLR are strongly predictive of primary patency failure (12 months after revascularization)
NLR [52]	Cohort retrospective, single center	PAD patients undergoing femoral endarterectomy	200	The association between preoperative NLR and 5-year mortality	>4	0.698	64.0	75.7	A high ratio was an independent factor associated with 5-year mortality
NLR [54]	Cohort prospective, single center	Patients with femoropopliteal arterial disease following DCB angioplasty	120	The relationship between postoperative NLR and restenosis in patients with femoropopliteal arterial disease following DCB angioplasty	>2.78	0.666	80.8	52.3	Postoperative NLR is independently associated with late restenosis
NLR [56]	Cohort retrospective, single center	PAD patients with femoropopliteal artery disease treated with DCBs	117	The relationship between preoperative NLR and 1-year restenosis after DCB for femoropopliteal artery disease	-	-	-	-	Baseline ratio before DCB can predict the risk of restenosis after surgery (OR = 1.47; 1.13–2.48)
NLR [60]	Cohort retrospective, single center	PAD patients undergoing PVI of femoropopliteal arteries	488	The association between preoperative NLR and clinical outcomes	>3	-	-	-	Elevated NLR is an independent predictor of decreased AFS (HR = 1.08; 1.05–1.11; *p* < 0.0001), decreased survival (HR = 1.09; 1.06–1.13; *p* < 0.0001) and freedom from major amputation survival through 4 years (HR = 1.06; 1.01–1.12; *p* = 0.01) Baseline NLR > 3 may be predictive of all-cause mortality and major amputation
NLR [61]	Cohort prospective, single center	CTLI patients	195	The association between the NLR and clinical outcomes in CTLI	≥8 ≥6	-	-	-	NLR has been associated with higher incidences of all-cause (*p* < 0.001) and cardiac-related mortality (adjusted HR: 5.286; 2.075–13.47, *p* < 0.001) MALEs (adjusted HR: 2.804; 1.292–6.088, *p* = 0.009)
NLR [62]	Cohort retrospective, single center	ALI patients with RC grade > I	210	The relationship between preoperative NLR and 30-day outcomes	>4.33	0.858	84.8	79.7	Baseline high NLR value was an independent predictor of amputation (OR: 11.09; 5.48–22.42; *p* < 0.0001), mortality (OR: 22.24; 9.61–51.47; *p* < 0.0001)
NLR [63]	Cohort retrospective, single center	PAD patients with Rutherford IIa or IIb ALI undergoing lower limb revascularization	345	The association between preoperative NLR and clinical outcomes	≥5.4	-	90.5	73.6	A higher pre-operative ratio is associated with 30-day death or amputation
PLR [62]	Cohort retrospective, single center	ALI patients with RC grade > I	210	The relationship between preoperative PLR and 30-day outcomes	>143.34	0.759	81.8	68.9	Baseline high PLR value was an independent predictor of amputation (OR: 8.97; 4.44–18.16; *p* < 0.0001), mortality (OR: 8.32; 3.90–17.73; *p* < 0.0001)
AISI [70]	Cohort retrospective, single center	Symptomatic PAD patients undergoing endovascular interventions	632	The relationship between preoperative AISI and the restenosis risk in SFA lesions	>489.64	0.623	57	56	High AISI is a biomarker for predicting the risk of restenosis (HR: 2.133; 1.508–3.017, *p* < 0.001)
SII [66]	Cohort retrospective, single center	PAD patients with lower extremity ASO	309	The associations between pretreatment SII and restenosis	≥357	0.715	-	-	SII is an independent predictor of restenosis
SIRI [69]	Cohort retrospective, single center	CLTI patients with Fontaine stage 4 undergoing successful EVT	168	The associations between SIRI and wound healing	-	0.840	-	-	SIRI predicts the potential for wound healing during in-hospital follow-up (aOR = 0.443; 0.313–0.625, *p* < 0.001)
CAR [71]	Cohort retrospective, single center	CLTI patientsundergoing EVT for BTK lesions	172	The association betweenpreprocedural CAR and long-term mortality in patients with CLTI	>4.33	0.771	80	66.7	The CAR is an independent predictor of all-cause mortality
HALP score [75]	Cohort retrospective, single center	Symptomatic PAD patients EVT		The HALP score’s relation to lesion severity and long-term mortality in PAD patients	-	0.736	-	-	The HALP score (HR, 0.087; 0.025–1.300; *p* < 0.001) independently predicted mortality

## Data Availability

The original contributions presented in this study are included in the article. Further inquiries can be directed to the corresponding author.

## Abbreviations

The following abbreviations are used in this manuscript:

ABI	Ankle-Brachial Index
AFS	Amputation-free survival
AISI	The Aggregate Index of Systemic Inflammation
ALE	Acute limb event
ALI	Acute limb ischemia
AMI	Acute myocardial infarction
ASCVD	atherosclerotic cardiovascular disease
ASO	Arteriosclerosis obliterans
AUC	Area under the curve
CAD	Coronary artery disease
CAR	C-reactive protein-to-albumin ratio
CLTI	Chronic limb-threatening ischemia
CRP	C-reactive protein
CVD	Cardiovascular disease
DAPT	Dual antiplatelet therapy
DCB	Drug-coated balloon
DPP4Is	Dipeptidyl Peptidase-4 Inhibitors
EVT	Endovascular therapy
FABPs	Fatty acid-binding proteins
Fib	Fibrinogen
FMD	Flow-mediated dilation
GAL-3	Galectine-3
GDF-15	Growth Differentiation Factor 15
GLP-1	Glucagon-like peptide-1
HALP score	Hemoglobin, Albumin, Lymphocyte, and Platelet score
HGMB-1	High-Mobility Group Box-1
hs-CRP	High-sensitivity C-reactive protein
IC	Intermittent claudication
IL	Interleukins
IL-10	Interleukin-10
IL-1β	Interleukin-1β
IL-27	Interleukin-27
IL-6	Interleukin-6
IL-8	Interleukin-8
IMT	Intima-media thickness
IPE	Icosapent ethyl
IPH	Intraplaque hemorrhage
LCN-2	Lipocalin-2
LDL	Low-density lipoproteins
LDL-C	Low-density lipoprotein cholesterol
LMR	Lymphocyte-to-Monocyte Ratio
Lp(a)	Lipoprotein(a)
MAC	Medial arterial calcification
MACEs	Major adverse cardiovascular events
MALEs	Major adverse limb events
MI	Myocardial infarction
MLR	Monocytes/Lymphocytes ratio
MPO	Myeloperoxidase
MPV	Mean platelet volume
NAFLD	Non-alcoholic fatty liver disease
NLR	Neutrophils/Lymphocytes ratio
OPG	Osteoprotegerin
PAD	Peripheral arterial disease
PCI	Percutaneous coronary intervention
PIV	Pan-Immune Inflammation Value
PLR	Platelets/Lymphocytes ratio
PTA	Percutaneous transluminal angioplasty
SFA	Superficial femoral artery
SGLT2Is	Sodium-glucose co-transporter 2 inhibitors
SII	Systemic immune-inflammation index
SIRI	The Systemic Inflammatory Response Index
siRNA	Small interfering RNA
sST2	Soluble Suppression of Tumorigenicity 2
TCFAs	Thin-Cap Fibroatheromas
TNF-α	Tumor necrosis factor-α
